# Supplementary material for: The role of restrictive abortion laws on modern contraceptive use in Sub Saharan Africa
Source: PLOS Glob Public Health. 2025 Jul 10;5(7):e0004875. doi: 10.1371/journal.pgph.0004875 (PMC12244480; doi:10.1371/journal.pgph.0004875)
Supplement: S1 Appendix — (DOCX) [file pgph.0004875.s001.docx]

**S1 Appendix. Flow chart for inclusion and exclusion criteria for countries in the study**
