# Supplementary material for: The role of restrictive abortion laws on modern contraceptive use in Sub Saharan Africa
Source: PLOS Glob Public Health. 2025 Jul 10;5(7):e0004875. doi: 10.1371/journal.pgph.0004875 (PMC12244480; doi:10.1371/journal.pgph.0004875)
Supplement: S2 Appendix — (DOCX) [file pgph.0004875.s002.docx]

**S2 Appendix. Country-level descriptive statistics, weighted n= 453,195**

| **Name of country** | **Year of DHS survey** | **Sample size (%)** |
| --- | --- | --- |
| Benin | 2017/2018 | 15,928 (3.5) |
| Burkina Faso | 2021 | 17,087 (3.9) |
| Burundi | 2016/2017 | 17,659 (3.8) |
| Cameroon | 2018 | 13,616 (3.0) |
| Chad | 2014/2015 | 17,719 (3.9) |
| Comoros | 2012 | 5,329 (1.2) |
| Republic of Congo | 2011/2012 | 10,819 (2.4) |
| Côte d’Ivoire | 2021 | 14,877 (3.3) |
| Ethiopia | 2016 | 15,683 (3.5) |
| Gabon | 2019/2021 | 9,910 (2.2) |
| Gambia | 2019/2020 | 11,865 (2.6) |
| Ghana | 2014 | 9,396 (2.1) |
| Guinea | 2018 | 10,874 (2.4) |
| Kenya | 2014 | 31,079 (6.9) |
| Lesotho | 2014 | 6,621 (1.5) |
| Liberia | 2019/2020 | 8,065 (1.8) |
| Madagascar | 2021 | 18,869 (4.2) |
| Malawi | 2015/2016 | 24,562 (5.4) |
| Mali | 2018 | 10,519 (2.3) |
| Mauritania | 2019/2021 | 15,714 (3.5) |
| Namibia | 2013 | 9,176 (2.0) |
| Niger | 2012 | 11,160 (2.5) |
| Nigeria | 2018 | 41,821 (9.2) |
| Senegal | 2019 | 15,688 (3.5) |
| Sierra Leone | 2019 | 15,574 (3.4) |
| South Africa | 2016 | 8,514 (1.9) |
| Tanzania | 2015/2016 | 13,266 (2.9) |
| Togo | 2013/2014 | 9,480 (2.1) |
| Uganda | 2016 | 18,506 (4.1) |
| Zambia | 2018 | 13,683 (3.0) |
| Zimbabwe | 2015 | 9,955 (2.2) |

n: Sample size; DHS: Demographic and Health Survey
