# Supplementary material for: The role of restrictive abortion laws on modern contraceptive use in Sub Saharan Africa
Source: PLOS Glob Public Health. 2025 Jul 10;5(7):e0004875. doi: 10.1371/journal.pgph.0004875 (PMC12244480; doi:10.1371/journal.pgph.0004875)
Supplement: S4 Appendix — (DOCX) [file pgph.0004875.s004.docx]

**S4 Appendix. Multilevel modeling for LARC/permanent contraceptive use**

| **Characteristics** | **Null model** | **Individual-level, aOR (95% CI)** | **Country-level, aOR (95% CI)** | **Full model, aOR (95% CI)** |
| --- | --- | --- | --- | --- |
| **Abortion law** |  |  |  |  |
| Broadly liberal |  |  | Ref. | Ref. |
| Moderately restrictive |  |  | 0.76 (0.71, 0.81)** | 0.60 (0.55, 0.66)** |
| Highly restrictive |  |  | 1.12 (1.05, 1.21)** | 1.00 (0.92, 1.09) |
| **Legislation that allows adolescents to access contraception** |  |  |  |  |
| No legislative support |  |  | Ref. | Ref. |
| Partial legislative support |  |  | 1.41 (1.32, 1.51) | 1.14 (1.06, 1.22)** |
| Full legislative support |  |  | 1.91 (1.79, 2.05)** | 2.30 (2.14, 2.48)** |
| **Model fit information** |  |  |  |  |
| ICC | 0.050 | 0.035 | 0.040 | 0.028 |
| AIC | 205993.9 | 154073.8 | 198971.7 | 150708.4 |
| BIC | 206015.8 | 154275.9 | 199058.6 | 150974.4 |

* = p value<0.05, ** <0.01. aOR: adjusted Odds Ratios; CI: Confidence Interval; ICC: Intra-Cluster Correlation; AIC: Akaike Information Criterion; BIC: Bayesian Information Criterion.

Individual-level model was controlled for age, place of residence, educational level, wealth index, religion, visit by FP worker, heard of FP in the media, health insurance coverage, and marital status.

Country-level model was controlled for duration of abortion law years and CHE as a % of GDP.

Full model was controlled for both individual and country level covariates.
