# Supplementary material for: The role of restrictive abortion laws on modern contraceptive use in Sub Saharan Africa
Source: PLOS Glob Public Health. 2025 Jul 10;5(7):e0004875. doi: 10.1371/journal.pgph.0004875 (PMC12244480; doi:10.1371/journal.pgph.0004875)
Supplement: S5 Appendix — (DOCX) [file pgph.0004875.s005.docx]

**S5 Appendix. Sensitivity analysis**

**Results from multilevel linear probability models for each outcome**

| **Characteristics** | **Modern contraceptive use, adjusted coefficients (95% CI)** | **LARC/permanent contraceptive use, adjusted coefficients (95% CI)** |
| --- | --- | --- |
| **Abortion law** |  |  |
| Broadly liberal | Ref. | Ref. |
| Moderately restrictive | -0.065 (-0.075, -0.056)** | -0.024 (-0.029, -0.018)** |
| Highly restrictive | -0.015 (-0.024, -0.006)* | 0.002 (-0.003, 0.007) |
| **Legislation that allows adolescents to access contraception** |  |  |
| No legislative support | Ref. | Ref. |
| Partial legislative support | 0.019 (0.011, 0.027)** | 0.010 (0.006, 0.015)** |
| Full legislative support | 0.064 (0.056, 0.072)** | 0.046 (0.041, 0.051)** |

* = p value<0.05, ** = <0.01. CI: Confidence Interval.

Each model was controlled for duration of abortion law years, CHE as a % of GDP, age, place of residence, educational level, wealth index, religion, visit by FP worker, heard of FP in the media, health insurance coverage, and marital status.
