# Supplementary material for: The role of restrictive abortion laws on modern contraceptive use in Sub Saharan Africa
Source: PLOS Glob Public Health. 2025 Jul 10;5(7):e0004875. doi: 10.1371/journal.pgph.0004875 (PMC12244480; doi:10.1371/journal.pgph.0004875)
Supplement: S6 Appendix — (DOCX) [file pgph.0004875.s006.docx]

**S6 Appendix. Sensitivity analysis**

**Multiple imputation for missing variables**

South Africa, Tanzania, Mauritania and Niger had no observations for religion leading to 10.9% missingness. Furthermore, South Africa, Gabon and Congo also had no observations for visited by a family planning worker contributing to 12.5% missingness. Health insurance coverage and access to family planning information via the media also had missingness of 7.8% and 3.6% respectively. Multiple imputation was therefore conducted to assess the robustness of the main results. Multivariable imputation by chained equations (MICE) was built for each of the two outcome variables; modern contraceptive use, and LARC/permanent contraceptive use. MICE is the recommended imputation of choice for categorical variables and was therefore appropriate for this analysis since both variables for imputation were categorical.

We specified 20 imputations for each of the two outcome variables. The primary exposures and all the control variables (including the weight, cluster, and strata variables) in each of the two models were specified accordingly. Results are presented in adjusted odds ratios.

**Results from multiple imputation datasets for each of the two outcomes**

| **Characteristics** | **Modern contraceptive use, aOR (95% CI)** | **LARC/permanent contraceptive use, aOR (95% CI)** |
| --- | --- | --- |
| **Abortion law** |  |  |
| Broadly liberal | Ref. | Ref. |
| Moderately restrictive | 0.65 (0.62, 0.68)** | 0.69 (0.65, 0.74)** |
| Highly restrictive | 0.95 (0.91, 0.99)* | 1.06 (0.99, 1.14) |
| **Legislation that allows adolescents to access contraception** |  |  |
| No legislative support | Ref. | Ref. |
| Partial legislative support | 1.06 (1.02, 1.11)** | 1.36(1.27, 1.45)** |
| Full legislative support | 1.61 (1.53, 1.69)** | 2.37 (2.21, 2.54)** |

* = p value<0.05, ** = <0.01. aOR: adjusted Odds Ratios; CI: Confidence Interval.

Each model was controlled for duration of abortion law years, CHE as a % of GDP, age, place of residence, educational level, wealth index, religion, visit by FP worker, heard of FP in the media, health insurance coverage, and marital status.
