# Supplementary material for: The role of restrictive abortion laws on modern contraceptive use in Sub Saharan Africa
Source: PLOS Glob Public Health. 2025 Jul 10;5(7):e0004875. doi: 10.1371/journal.pgph.0004875 (PMC12244480; doi:10.1371/journal.pgph.0004875)
Supplement: S7 Appendix — (DOCX) [file pgph.0004875.s007.docx]

**S7 Appendix. Sensitivity analysis**

**Sample restricted to only married women for each of the two outcomes**

| **Characteristics** | **Modern contraceptive use, aOR (95% CI)** | **LARC/permanent contraceptive use, aOR (95% CI)** |
| --- | --- | --- |
| **Abortion law** |  |  |
| Broadly liberal | Ref. | Ref. |
| Moderately restrictive | 0.50 (0.45, 0.54)** | 0.68 (0.61, 0.75)** |
| Highly restrictive | 0.81 (0.76, 0.86)** | 1.02 (0.94, 1.11) |
| **Legislation that allows adolescents to access contraception** |  |  |
| No legislative support | Ref. | Ref. |
| Partial legislative support | 0.96 (0.90, 1.02) | 1.34 (1.24, 1.45)** |
| Full legislative support | 1.83 (1.72, 1.95)** | 2.72 (2.51, 2.95)** |

* = p value<0.05, ** = <0.01. aOR: adjusted Odds Ratios; CI: Confidence Interval.

Each model was controlled for duration of abortion law years, CHE as a % of GDP, age, place of residence, educational level, wealth index, religion, visit by FP worker, heard of FP in the media, health insurance coverage, and marital status.
