# Supplementary material for: The role of restrictive abortion laws on modern contraceptive use in Sub Saharan Africa
Source: PLOS Glob Public Health. 2025 Jul 10;5(7):e0004875. doi: 10.1371/journal.pgph.0004875 (PMC12244480; doi:10.1371/journal.pgph.0004875)
Supplement: S8 Appendix — (DOCX) [file pgph.0004875.s008.docx]

**S8 Appendix. Sensitivity analysis**

**Results from multivariate logistic regression mixed effects models**

| **Characteristics** | **Modern contraceptive use, aOR (95% CI)** | **LARC/permanent contraceptive use, aOR (95% CI)** |
| --- | --- | --- |
| **Abortion law** |  |  |
| Broadly liberal | Ref. | Ref. |
| Moderately restrictive | 0.62 (0.58, 0.66)** | 0.60 (0.55, 0.66)** |
| Highly restrictive | 0.88 (0.83, 0.93)** | 1.02 (0.94, 1.11) |
| **Legislation that allows adolescents to access contraception** |  |  |
| No legislative support | Ref. | Ref. |
| Partial legislative support | 1.03 (0.98, 1.09) | 1.16 (1.08, 1.25)** |
| Full legislative support | 1.54 (1.46, 1.62)** | 2.30 (2.12, 2.49)** |

* = p value<0.05, ** = <0.01. aOR: adjusted Odds Ratios; CI: Confidence Interval.

Each model was controlled for duration of abortion law years, CHE as a % of GDP, age, place of residence, educational level, wealth index, religion, visit by FP worker, heard of FP in the media, health insurance coverage, and marital status.
